# Oxidative Stress-Induced Hypertension of Developmental Origins: Preventive Aspects of Antioxidant Therapy

**DOI:** 10.3390/antiox11030511

**Published:** 2022-03-07

**Authors:** You-Lin Tain, Chien-Ning Hsu

**Affiliations:** 1 Department of Pediatrics, Kaohsiung Chang Gung Memorial Hospital and Chang Gung University College of Medicine, Kaohsiung 833, Taiwan; tainyl@cgmh.org.tw; 2 Institute for Translational Research in Biomedicine, Kaohsiung Chang Gung Memorial Hospital and Chang Gung University College of Medicine, Kaohsiung 833, Taiwan; 3 Department of Pharmacy, Kaohsiung Chang Gung Memorial Hospital, Kaohsiung 833, Taiwan; 4 School of Pharmacy, Kaohsiung Medical University, Kaohsiung 807, Taiwan

**Keywords:** antioxidant, hypertension, nitric oxide, asymmetric dimethylarginine, oxidative stress, developmental origins of health and disease (DOHaD), reactive oxygen species, renin-angiotensin system

## Abstract

Hypertension remains the leading cause of disease burden worldwide. Hypertension can originate in the early stages of life. A growing body of evidence suggests that oxidative stress, which is characterized as a reactive oxygen species (ROS)/nitric oxide (NO) disequilibrium, has a pivotal role in the hypertension of developmental origins. Results from animal studies support the idea that early-life oxidative stress causes developmental programming in prime blood pressure (BP)-controlled organs such as the brain, kidneys, heart, and blood vessels, leading to hypertension in adult offspring. Conversely, perinatal use of antioxidants can counteract oxidative stress and therefore lower BP. This review discusses the interaction between oxidative stress and developmental programming in hypertension. It will also discuss evidence from animal models, how oxidative stress connects with other core mechanisms, and the potential of antioxidant therapy as a novel preventive strategy to prevent the hypertension of developmental origins.

## 1. Introduction

Hypertension is the number one risk factor for global deaths, affecting one in three adults across the world [1,2]. Significant interest has recently focused on the fact that the origins of hypertension can begin in early life [3,4,5]. Now this concept, based on observing that the developing fetus being exposed to adverse conditions in utero increases the risk for chronic diseases happening later in life, has been termed as the “developmental origins of health and disease (DOHaD)” [6]. The hypertension of developmental origins can be programmed by a number of modifiable environmental risk factors [7,8,9,10], especially those linked to maternal nutrition. Notably, the DOHaD concept, besides determining the early-life risk for the developmental programming of hypertension, offers a novel way to prevent hypertension by reprogramming therapy [11]. By switching therapy from adulthood to early life prior to illness onset, we have the potential to reverse adverse programming processes that would lead to hypertension. Accordingly, one may assume that early reprogramming therapy serves as an innovative strategy to reduce the burden of hypertension globally.

A growing body of evidence shows that oxidative stress plays a pivotal role in the pathogenesis of hypertension [12,13,14,15]. The overproduction of deleterious reactive oxygen and nitrogen species (ROS and RNS) that overwhelm the cellular antioxidant capacity results in pathogenic oxidative stress [16]. Experimental evidence supports that a ROS/nitric oxide (NO) disequilibrium under oxidative stress favors oxidation reactions involved in major blood pressure (BP)-controlled organs such as the brain, kidneys, heart, and blood vessels, underpinning the development of hypertension [12,13,14,15]. Conversely, treatment with antioxidants has been suggested to lower oxidative stress and therefore BP [16,17].

Although cumulative evidence has shown the impact of oxidative stress and antioxidant therapy in established hypertension [12,13,14,15,16,17,18], less attention has been paid to their implications in the hypertension of developmental origins. Therefore, the purpose of the current review is to highlight the best available evidence on the interaction between oxidative stress and the developmental programming of hypertension. In this review, attempts will be made to discuss the role of oxidative stress in the hypertension of developmental origins, its associations with the core mechanisms of developmental programming behind hypertension, and the potential of antioxidant therapy as a novel preventive strategy for the hypertension of developmental origins.

The PubMed/MEDLINE database was used to identify related peer-reviewed journal articles published in English between January 1980 and December 2021. We used the following search keywords: “antioxidants”, “hypertension”, “blood pressure”, “developmental programming”, “DOHaD”, “free radicals”, “offspring”, “progeny”, “mother”, “prenatal”, “nitric oxide”, “oxidative stress”, “pregnancy”, “reprogramming”, “reactive oxygen species”, “reactive nitrogen species”, and “vitamin”. Additional studies were selected based on references from eligible articles.

## 2. Oxidative Stress and Hypertension

### 2.1. ROS/NO Disequilibrium

Oxidative stress has been characterized as a disturbance in the ROS/NO balance, with the notion that ROS and RNS damage biological molecules [16]. Both ROS and RNS collectively constitute free radicals and non-radical reactive species. ROS include free radicals such as superoxide anion (O_2_^−^) and hydroxyl anion (OH^−^) as well as non-radical molecules such as hydrogen peroxide (H_2_O_2_). The main enzyme sources for superoxide are nicotinamide adenine dinucleotide phosphate (NADPH) oxidases, xanthine oxidase, cyclooxygenases, lipoxygenases, and the mitochondrial respiration chain [19]. The superoxide anion radical initiates a cascade of reactions resulting in the generation of other ROS. RNS include peroxynitrite (ONOO-) and its reaction products, such as nitrogen dioxide (NO_2_). Much of RNS-dependent cytotoxicity resides on peroxynitrite, formed by high levels of NO and superoxide [20]. Conversely, our body has various antioxidants to ameliorate the harmful effects of ROS/RNS. This antioxidant machinery has two arms: (1) enzymatic components consisting of superoxide dismutase (SOD), catalase, glutathione peroxidase (GPx), and glutathione reductase, and (2) non-enzymatic antioxidants such as glutathione (GSH) and vitamins [21]. 

At an optimal level, NO physiologically functions as a gasotransmitter and as a vasodilator. NO is generated via a reaction involving the conversion of l-arginine to l-citrulline by a family of NO synthases (NOSs). There are three isoforms of NOS: neuronal NOS (nNOS), endothelial NOS (eNOS), and inducible NOS (iNOS). Asymmetric dimethylarginine (ADMA) is an endogenous competitive inhibitor of NOS [22]. In the presence of high ADMA levels, NOS isoenzymes become uncoupled to produce peroxynitrite, further contributing to the reduced NO bioavailability and increased oxidative stress [23]. Considering both ROS and NO behave as BP modulators, maintenance of the ROS/NO balance is required for strict BP control.

### 2.2. Oxidative Stress and Hypertension

The heart, kidneys, brain, and blood vessels are prime organs involved in the regulation of BP. Under physiological conditions, the maintenance of normal BP requires the coordinated interplay of several regulatory players, including the renin–angiotensin system (RAS), natriuretic peptides, sodium excretion, the endothelium, and the sympathetic nervous system [24]. Oxidative stress not only contributes to BP-controlled organ dysfunction and remodeling through oxidative damage but also impairs coordinated regulatory systems, leading to hypertension [12,13,14,15]. Each organ system will be discussed in turn.

#### 2.2.1. Cardiovascular System 

Blood pressure can be determined by cardiac output and the resistance of blood vessels. Endothelial cells are important constituents of blood vessels that play key roles in cardiovascular homeostasis [25]. Endothelial dysfunction is characterized by reduced vasodilation, pro-thrombotic settings, and a pro-inflammatory state, which together are implicated in the pathophysiology of hypertension. These events can be mediated by excessive ROS formed by vascular walls [26]. The vasculature is a major source of NADPH-oxidase-derived ROS, which has a vital role in vascular damage under pathological conditions [26]. Additionally, several endothelium-derived vasoconstricting factors, such as angiotensin II (Ang II), endothelin, urotensin II, vasoconstrictor prostaglandins, and thromboxane A2, can be released by endothelial cells and contribute to the vasoconstrictor effects.

Conversely, reduced NO bioavailability, a well-known endothelium-derived relaxing factor, is considered a hallmark of endothelial dysfunction [27]. The eNOS-derived NO is responsible for vasodilation within the endothelium. Loss of l-arginine and cofactor tetrahydrobiopterin (BH4) leads to the uncoupling of eNOS, in which the enzyme produces superoxide anion rather than nitric oxide, further generating peroxynitrite to induce vascular pathogenesis. Likewise, ADMA can uncouple NOS isoenzymes to form superoxide, contributing to endothelial dysfunction [28]. The activation of oxidative stress not only disturbs the balance between vasodilators and vasoconstrictors affecting vascular tone but also ultimately leads to vascular dysfunction, abnormalities of vessels, atherosclerosis, arterial stiffness, and vascular remodeling [29]. 

Collectively, there is significant evidence indicating that vascular oxidative stress induces vasoconstriction and remodeling of blood vessels, contributing to the development of hypertension [14].

#### 2.2.2. Renal System

Several lines of evidence clearly show that renal oxidative stress is a key factor in the development of hypertension [30]. Increased ROS in the kidneys have been found in various hypertensive models, such as spontaneously hypertensive rat (SHR), stroke-prone SHR, DOCA-salt hypertension rat, Dahl salt-sensitive rat, and 5/6 nephrectomy rat [31,32,33,34,35]. In addition, extracellular SOD knockout mice have been found to display increased renal oxidative stress and develop hypertension [36]. Additionally, knockout of nNOS in macula densa causes the elevation of BP in mice [37]. Increased oxidative stress markers, such as 8-hydroxydeoxyguanosine (8-OHdG) expression in the kidneys, have been reported in various models of established and programmed hypertension, such as SHR [38], Dahl salt-sensitive rat [39], maternal high-fat diet [40], and maternal high-fructose diet [41]. Another line of evidence comes from antioxidant therapy for the management of oxidative-stress-induced hypertension [12,18,42,43,44]. Several antioxidants, such as vitamin E [35], melatonin [45], and *N*-acetylcysteine [46], have shown a beneficial effect on hypertension coinciding with a reduction in renal oxidative stress.

#### 2.2.3. Central Nervous System

The central nervous system (CNS) controls regional sympathetic outflow to target organs (e.g., heart and kidneys) through the integration of neural signals from autonomic brain networks, input from circulating factors, and reflex influences [15]. Results from human and animal studies reveal that overexcitation of the sympathetic nervous system plays a key role in the pathogenesis of hypertension [15]. Neurons that generate central sympathetic vasomotor outflow reside in the rostral ventrolateral medulla (RVLM) of the brain stem [47]. All ROS- and NO-generating enzymes are present in the RVLM [48]. ROS increase sympathoexcitatory inputs to RVLM neurons, whereas iNOS-derived NO promotes sympathoinhibition [49]. Accordingly, a shift from ROS to NO in the RVLM underpins the succession of increase to decrease in sympathetic vasomotor tone that is responsible for the development of hypertension [48,49]. 

An ever-growing body of evidence supports that increased brain stem oxidative stress contributes to the generation of hypertension. Increased NADPH-oxidase expression and activity has been demonstrated in the RVLM of SHR [50], while the knockdown of its subunits blunts the enhanced central sympathetic outflow and protects against hypertension [51]. Reduced NO in the RVLM, in conjunction with augmented Ang-II-induced oxidative stress also contributes to sympathoexcitation in hypertension [52]. Conversely, interventions that quench ROS have been reported to reduce brain oxidative stress and prevent neurogenic hypertension. The microinjection of tempol [53], CoQ10 [54], or catalase [55] into the RVLM has been reported to blunt hypertension and inhibit brain oxidative stress.

#### 2.2.4. The Regulatory Hormones

In view of the introduction of renal sympathetic denervation into clinical medicine that has demonstrated marked reductions in BP in patients with resistant hypertension [56], there has been interest in interorgan crosstalk involving various regulatory hormones contributing to hypertension that more closely reflects the human condition [57,58,59]. These regulatory hormones include, but are not limited to, the RAS [59], cytokines [60], norepinephrine [61], atrial natriuretic peptide [61], endothelin [62], urotensin II [63], prostaglandins [57], melatonin [64], cortisol [65], hydrogen sulfide [66], and gut-microbiota-derived metabolites [67].

**Figure 1:**
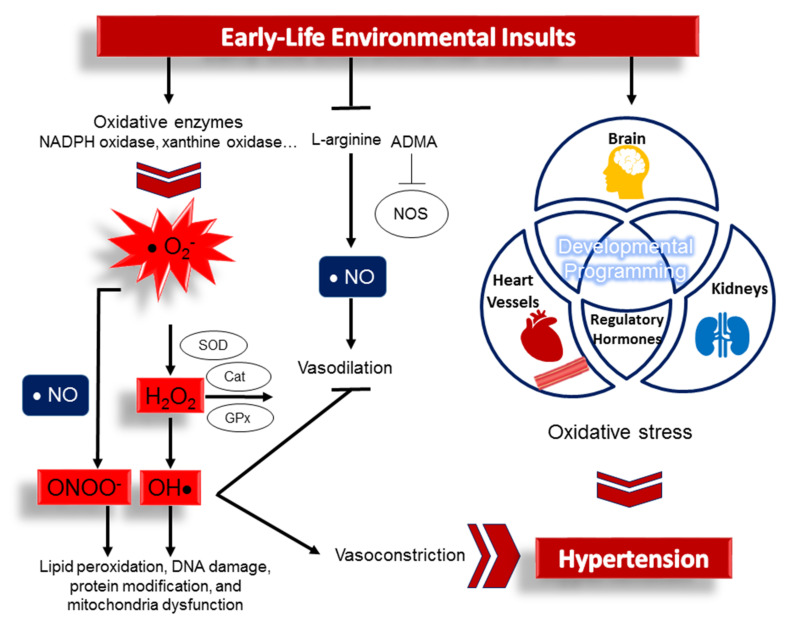
Schema outlining how early-life environmental insults induce hypertension in later life via oxidative stress programming of various organ systems and regulatory hormones. Early-life insults cause an increase in reactive oxygen species (ROS) and a decrease in nitric oxide (NO). ROS are derived from enzymes that produce superoxide radical (O_2_^−^) intracellularly, such as NADPH oxidase and xanthine oxidase. Excessive ROS can be offset by the action of antioxidant enzymes. The components of the antioxidant defense are superoxide dismutase (SOD), glutathione peroxidase (GPx), catalase (Cat), etc. Nitric oxide synthase (NOS) catalyzes l-arginine to produce NO. NO mediates vasodilation and opposes vasoconstrictor effects driven by ROS. Uncoupled NOS produces superoxide, which scavenges NO, leading to peroxynitrite (ONOO^−^) formation. Oxidative stress is a condition where ROS overwhelm the antioxidant system, leading to cellular injury in the form of damaged DNA, lipids, and proteins. During development, oxidative stress triggers the developmental programming of prime organs involved in the regulation of blood pressure (i.e., heart, kidneys, brain, and blood vessels) and regulatory hormones, leading to hypertension in later life.

Together these observations suggest that oxidative stress could be a core mechanism involved in interorgan communication via various regulatory hormones contributing to hypertension. However, although these studies highlight that oxidative stress correlates with established hypertension, its role in the hypertension of developmental origins has received little attention from researchers. A summary of the interaction between maternal insults implicated in oxidative stress and the prime organ system involved in the developmental programming of hypertension is depicted in Figure 1.

## 3. Oxidative-Stress-Related Hypertension of Developmental Origins

### 3.1. Oxidative Stress during Pregnancy

Fetal oxygen requirements vary at various stages of pregnancy [68]. During the first trimester, fetal oxygen levels are low. However, increasing oxygen need happens, during the second and third trimesters, for the formation of fetal-placental circulation and the rapid gain of fetal weight [69]. Oxidative damage occurs in a compromised pregnancy owing to the failure of defensive antioxidant mechanisms in responding to excessive ROS and RNS [70]. Many adverse conditions in pregnancy result in increased oxidative stress, including obesity, diabetes, preeclampsia, maternal smoking, and intrauterine growth retardation (IUGR) [71]. Accordingly, oxidative stress adversely affects the developing fetus, resulting in adult disease in later life [10,72]. 

### 3.2. Evidence from Human Studies

The association between the hypertension of developmental origins and low birth weight (LBW) was first highlighted by David Barker and colleagues in the late 1980s [73]. Another important observation from the Dutch Hunger Winter Study was that maternal undernutrition has lasting, negative effects on offspring health, including hypertension [74]. Additionally, several mother-child cohorts provide important support for the developmental programming of hypertension. A variety of early-life risks associated with offspring hypertension have been acknowledged, including maternal smoking [75], maternal obesity [76], low vitamin D consumption [77], gestational hypertension [78], IUGR [79], and short-term breastfeeding [80]. 

Although human observational studies provide relevant evidence correlating early-life factors with the developmental programming of hypertension, the direct cause-and-effect relationships between oxidative stress and offspring hypertension that drive programming processes cannot be established and therefore it is difficult to identify a potential reprogramming strategy. 

### 3.3. Evidence from Animal Studies

In recent years, our understanding of the molecular mechanisms behind the hypertension of developmental origins has grown by using animal models [81,82]. These mechanisms contain oxidative stress, reduced nephron number, aberrant activation of the RAS, dysregulated nutrient-sensing signals, gut microbiota dysbiosis, and so on [9,10,11,81]. Among them, oxidative stress has a crucial role and is closely interconnected to other core mechanisms behind the hypertension of developmental origins.

The present review is limited to adverse early-life insults beginning in pregnancy and lactation, with a focus on the oxidative-stress-related hypertension of developmental origins. Table 1 shows that developmental programming of hypertension is related to oxidative stress in various organ systems, which leads to significant changes in offspring BP [46,83,84,85,86,87,88,89,90,91,92,93,94,95,96,97,98,99,100,101,102,103,104,105,106,107,108,109,110,111,112,113,114,115,116,117,118,119]. Diverse environmental insults can induce the oxidative-stress-related hypertension of developmental origins. These insults can be grouped into maternal nutritional imbalance [83,84,85,86,87,93,94,95,96,97,100,108,111,112], maternal illness [46,88,92,98,99], pregnancy complications [107,109,110,113,117,118,119], exposure to environmental chemicals [105,106,114], and maternal medication [89,90,91,115,116].

Maternal nutritional insults are the leading causes of the oxidative-stress-related hypertension of developmental origins in animal models. Under- and over-nutrition can both induce nutritional programming [120]. These nutritional risk factors include calorie restriction [83,84,85,86], protein restriction [87], and a diet high in fructose [93,94,95,96], fat [100,108], salt [111,112], or methyl-donors [97]. Another factor interfering with oxidative stress programming is maternal illness. Maternal diabetes [89,90,91], preeclampsia [92], CKD [98,99], and hypertension [109,110] have been reported to induce oxidative stress and the hypertension of developmental origins concurrently. Additionally, pregnancy complications such as reduced uterine perfusion [107], inflammation [113], and hypoxia [117,118,119] are also relevant to oxidative-stress-related programmed hypertension. Offspring hypertension can also be programmed by dams exposed to environmental chemicals, such as 2,3,7,8-tetrachlorodibenzo-p-dioxin (TCDD) [105], bisphenol A [106], and di-n-butyl phthalate [114]. Moreover, medication use such as glucocorticoid can program the hypertension of developmental origins [89,90,91,115,116].

Rats are the most commonly used species. Other species such as chicken [118] and sheep [115,116,119] have also been used to evaluate the hypertension of developmental origins. Considering that rats reach sexual maturity at 8–10 weeks of age, and in adulthood each month of the life of a rat is equivalent to 3 human years [121], Table 1 lists the timing of developing hypertension ranging from 8 weeks to 6 months of age in rats, which corresponds to humans from childhood to early adulthood.

### 3.4. Mechanisms Underpinning Oxidative Stress in Hypertension of Developmental Origins

There are several oxidative-stress-mediated mechanisms involved in the pathogenesis of programmed hypertension, including increased ROS [91,95,110,114,116], increased ROS-producing enzyme expression [86,94,101,107,109,112], decreased antioxidant capabilities [91,100,101,112], an impaired ADMA–NO pathway [83,84,85,88,89,91,92,93,98,99,102,103,104,105,106,116,118,119], increased peroxynitrite [85,90,109,111], and increased oxidative damage [46,83,84,87,90,93,94,96,97,98,99,100,103,104,105,106,107,108,109,112,115,117]. 

3-nitrotyrosine (3-NT) is a marker of oxidative stress formed due to the nitration of protein-bound and free tyrosine residues by reactive peroxynitrite molecules [122]. Table 1 shows that increased 3-NT in the vessels [85,111], kidneys [90], and brain [109] is associated with the hypertension of developmental origins.

Prior work indicates that ADMA-related NO–ROS imbalance in early life induces adulthood hypertension [123]. Several studies support the notion that ADMA is a key risk factor related to oxidative stress programming in various programming models, such as caloric restriction [83,84], diabetes [89], preeclampsia [92], maternal CKD [98,99], combined dexamethasone and TCDD exposure [105], prenatal bisphenol A exposure and high-fat diet [106], and high-salt diet [111]. NO deficiency in the vessels [85] and kidneys [102] is also relevant to the hypertension of developmental origins. 

Several frequently used markers of lipid peroxidation have been used to detect oxidative damage in models of programmed hypertension, including F_2_-isoprostanes [46,87], thiobarbituric acid reactive substances (TBARS) [90], malondialdehyde (MDA) [94,100,112], and 4-hydroxynonenal (4-NHE) [115]. As shown in Table 1, the hypertension of developmental origins induced by various maternal insults is associated with lipid peroxidation in the kidneys [46,87,113], vessels [90,112], and brain [94]. In addition, 8-hydroxydeoxyguanosine (8-OHdG) is the most frequently detected and studied oxidized nucleoside of DNA [124]. Maternal caloric restriction [83,84], a high-fructose diet [93], a maternal methyl-deficient or donor-rich diet [97], CKD [98,99], prenatal dexamethasone exposure [103,104,105], combined dexamethasone and TCDD exposure [105], prenatal bisphenol A exposure and high-fat diet [106], and high-fat diet [108] have been shown to give rise to programmed hypertension in the presence of increased 8-OHdG expression.

It is noteworthy that most studies have mainly focused on the renal and cardiovascular systems; rather less attention has been paid to oxidative stress programming on other organ systems, including the brain [94,109,115], spleen [95], and adrenal glands [101].

### 3.5. Oxidative-Stress-Induced Renal Programming

During development, the fetal kidney is susceptible to adverse early-life events, leading to changes in structure and function, namely renal programming [125]. Renal programming is the most commonly studied mechanism behind the hypertension of developmental origins [4,5,11]. A reduced nephron number can develop during pregnancy through childhood to later life in different animal models of renal programming [126]. Maternal insults need only last for 1–2 days to impair nephrogenesis, resulting in a permanent reduction in the nephron number [127]. Accordingly, the main phenotype of renal programming associated with a reduced nephron number is hypertension [126]. 

An impaired ADMA–NO pathway is tightly linked to oxidative stress in determining renal programming [123]. NO deficiency in pregnancy induced by NOS inhibitor N^G^-nitro-l-arginine methyl ester (L-NAME) caused renal programming, coinciding with increased oxidative stress in adult offspring [46]. Additionally, maternal NO deficiency was able to modify more than 2000 renal transcripts in a 1-day-old offspring kidney. It has been found that several genes belonging to the RAS and arachidonic acid metabolism pathway contribute to the pathogenesis of programmed hypertension [46]. In a prenatal dexamethasone exposure model [102,127], offspring rats developed hypertension coinciding with a reduced nephron number, increased plasma ADMA levels, and reduced renal NO production. Likewise, the links between oxidative stress and a reduced nephron number have been reported in a caloric restriction model [83] and a streptozotocin-induced diabetes model [89]. Moreover, our prior work has demonstrated that ADMA can impair nephrogenesis [89]. Metanephroi grown in 2 or 10 µM ADMA displayed reduced nephron numbers in a dose-dependent manner [89]. When we treated cultured metanephroi with 10 µM ADMA, the next-generation sequencing (NGS) analysis identified 1221 differential expressed genes [128]. Among them, *Ephx2*, *Avpr1a*, *Npy1r*, *Hba2*, and *Hba-a2*, have been linked to programmed hypertension in other models [129,130]. Together, these observations support the notion that oxidative-stress-induced renal programming contributes to the hypertension of developmental origins.

### 3.6. Oxidative-Stress-Induced Cardiovascular Programming

The fetal cardiovascular system is similar to the developing kidneys with great vulnerability to adverse in utero conditions [131]. Oxidative stress may mediate developmental plasticity in the CV system with structural and functional changes during the organogenesis of the heart–vascular system, leading to endothelial dysfunction, a stiffer vascular tree, fewer cardiomyocytes, and small coronary arteries, through cardiovascular programming [131,132,133]. Table 1 documents several cardiovascular programming models that are relevant to oxidative stress, including maternal caloric restriction [85,86], diabetes [91], high-salt diet [111,112], and prenatal hypoxia exposure [118,119]. 

In the maternal caloric restriction model, offspring displayed high BP accompanied with a decrease in NOS activity in the microvessels and increased cardiac xanthine-oxidase expression [85,86]. Another study showed that adult offspring born to diabetic dams developed hypertension related to an increased ROS level and decreased SOD expression and NO bioavailability in mesenteric arteries [91]. Moreover, developmental hypoxia has been reported to impair the NO pathway and endothelial function, consequently programming hypertension in the adult offspring of chickens and sheep [118,119]. In rats and mice, cardiovascular maturation continues past birth, becoming completed by the second week postnatally. Unlike rodents, chickens and sheep share similar temporal windows of precocial cardiovascular development and maturation with humans [134]. Therefore, cardiovascular data derived from these two models provide a useful translation to the human situation.

In addition, endothelium-dependent hyperpolarization (EDH), a dominant vasodilator in resistance arteries, is also involved in the regulation of BP. Considering that oxidative stress impairs EDH during hypertension in some vessels [135], reduced EDH might contribute to the oxidative-stress-induced hypertension of developmental origins. Indeed, a previous study has shown that offspring exposed to a high-fat diet display reduced EDH and BP elevation concurrently, despite the mechanisms of oxidative stress remaining undetermined [136].

### 3.7. Other Mechanisms Related to Oxidative Stress Programming

**Figure 2:**
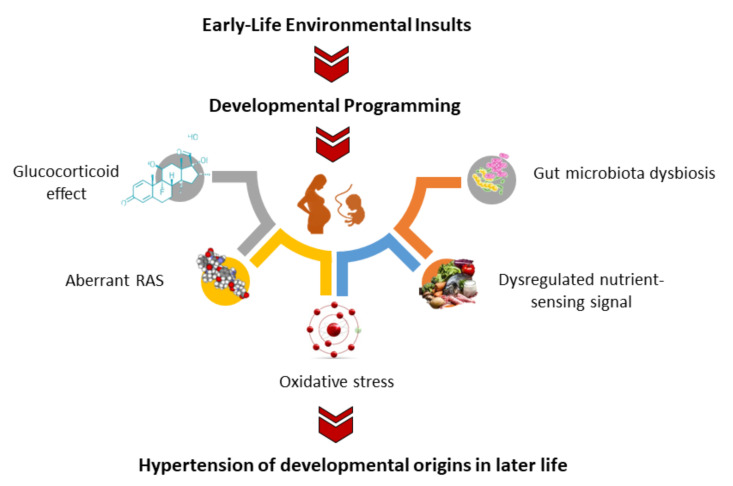
Oxidative stress and possible molecular pathways linked to the hypertension of developmental origins.

Considering a wide spectrum of early-life insults create similar outcomes (i.e., hypertension) in adult offspring, it is logical to think that common mechanisms contribute to the hypertension of developmental origins. In addition to oxidative stress, several mechanisms have been proposed, including glucocorticoid effect, aberrant RAS, dysregulated nutrient-sensing signals, gut microbiota dysbiosis, etc. [4,5,10,11,59,81]. Among them, oxidative stress plays a decisive role and is tightly interconnected to other core molecular pathways involved in the hypertension of developmental origins (Figure 2).

First, several studies have linked increased fetal glucocorticoid exposure to the developmental programming of hypertension in adult offspring [101,102,103,104,105,115,116]. One previous study reported that programmed male offspring exhibited reduced antioxidant glutathione peroxidase 1 (Gpx1) expression and increased NADPH-oxidase expression in the adrenal glands [101]. Second, the aberrant activation of the RAS is a well-known mechanism underlying the hypertension of developmental origins [59]. In a maternal hypertension model, offspring hypertension was found to be related to increased expression of angiotensin II type 1 receptor (AT1R) and oxidative-stress-related protein in the brain [109]. Conversely, an early blockade of the RAS by renin inhibitor aliskiren mitigates increases in ADMA and restores NO bioavailability, contributing to the decrease in BP in young spontaneously hypertensive rats (SHRs) [137].

Dysregulated nutrient-sensing signals are also involved in the development of hypertension [132]. In a maternal high-fructose model, the dysfunction of AMP-activated protein kinase (AMPK)-regulated AT1R expression and sirtuin 1 (SIRT1)-mediated mitochondrial biogenesis coincided with increased oxidative stress in RVLM, which in turn increased sympathetic activity and BP in offspring [94]. Besides, a maternal high-fructose diet was found to decrease expression of AMPK, SIRT4, and peroxisome proliferator-activated receptors (PPARs) in offspring kidneys [96].

Furthermore, recent evidence indicates that early development of the gut microbiota influences the development of hypertension [138,139]. Data from several animal models indicate that the interactions between gut microbiota dysbiosis and oxidative stress may contribute to the pathogenesis of programmed hypertension, such as maternal CKD [98], high-fructose diet [96], and high-fat diet [140] models.

## 4. Antioxidants as Reprogramming Strategies

Concerning our advanced understanding of the DOHaD concept, it turns out that a therapeutic approach can be started earlier, even before hypertension occurs, by so-called reprogramming [11]. In view of the fact that oxidative stress plays a critical role in the hypertension of developmental origins, it is reasonable to assume that antioxidant therapy would be an appropriate reprogramming strategy to protect offspring against programmed hypertension. This section discusses the reprogramming role of natural and synthetic antioxidants that have demonstrated an ability to participate in the main redox reactions and prevent the hypertension of developmental origins.

**Figure 3:**
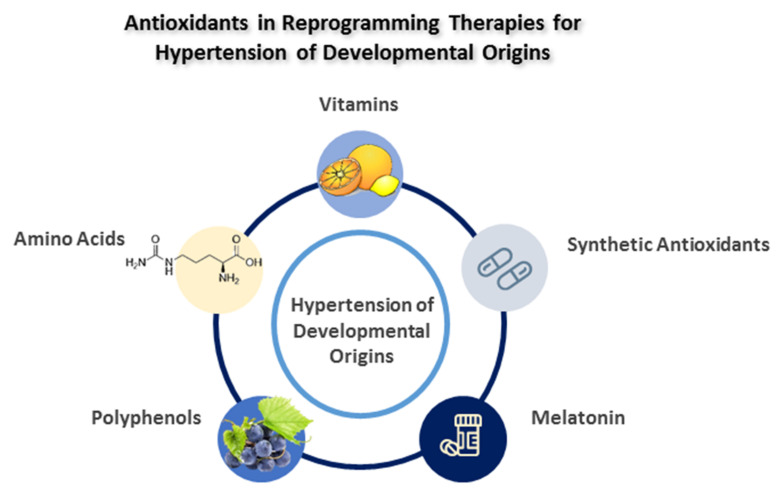
Schema outlining the potential antioxidants as a reprogramming strategy to prevent the hypertension of developmental origins.

There are two groups of antioxidants: enzymatic and non-enzymatic antioxidants [141]. Non-enzymatic antioxidants are classified as natural and synthetic antioxidants [142], which could be endogenous and exogenous. Examples of natural non-enzymatic antioxidants are glutathione, carotenoids, flavonoids, polyphenols, and vitamins A, C, and E [141]. A variety of plant materials are known to be sources of natural non-enzymatic antioxidants, such as vegetables, seeds, nuts, and fruits. As reviewed elsewhere [43], several natural antioxidants such as amino acids, vitamins, melatonin, and resveratrol have shown benefits for the prevention of developmental hypertension. Apart from natural antioxidants, several synthetic antioxidants have also been implemented in animal models of programmed hypertension. The potential antioxidants used as reprogramming therapies for the hypertension of developmental origins are illustrated in Figure 3. Each antioxidant will be discussed in turn.

### 4.1. Vitamins

Two of the most commonly used antioxidants are vitamins C and E. Vitamin C is a potent water-soluble antioxidant, possessing an ability to quench ROS. Vitamin E is a lipid-soluble antioxidant, which can inhibit NADPH oxidase, cyclooxygenase, and lipoxygenase [143]. Perinatal use of vitamin C or E, alone or combined with other antioxidants, has been shown to prevent the development of hypertension in later life [144,145,146]. To date, two studies have showed that maternal vitamin C or E supplementation prevented hypertension programmed by prenatal hypoxia in adult sheep or by maternal inflammation in adult rats, respectively [113,119]. 

### 4.2. Amino Acids

l-arginine is the substrate of NOS isoenzymes and l-citrulline is the main precursor of l-arginine [147]. Considering NO deficiency is a core pathogenetic mechanism behind hypertension of developmental origins, these two amino acids have been studied to ameliorate offspring hypertension in later life [102,103,104]. 

Although perinatal supplementation of l-arginine has shown benefits on intrauterine growth retardation in various models of developmental programming [148], whether the use of l-arginine in pregnancy will also help prevent the hypertension of developmental origins awaits further elucidation. l-arginine can be converted from l-citrulline in the kidneys [149]. Oral l-citrulline supplementation has been used to enhance l-arginine production and bypass hepatic metabolism to raise NO levels [149]. So far, maternal l-citrulline supplementation has been reported to protect adult rat offspring against oxidative stress programming, including in models of maternal caloric restriction [83], streptozotocin-induced diabetes [89], and prenatal dexamethasone exposure [102]. 

Although there are other amino acids (e.g., l-taurine and l-cysteine) showing reprogramming potentials for the hypertension of developmental origins [9], the relationships between their beneficial effects and oxidative stress remain largely unclear.

### 4.3. Polyphenols

Polyphenols are the widespread phytochemical antioxidants in food [150]. Studies have demonstrated the valuable effect of polyphenols in the control of oxidative stress by acting as free radical scavengers, stimulators of antioxidant enzymes, NOS activators, and metal chelators [150,151]. Accordingly, polyphenols enhanced vascular endothelial function, resulting in antihypertensive effects [152]. 

Resveratrol is a commonly used polyphenol as a nutritional supplement [153]. One of the most important ways in which resveratrol reduces ROS levels is via inhibiting NADPH oxidase [154]. Additionally, resveratrol is able to augment NOS expression, increase glutathione level, and enhance expression of antioxidant enzymes [154]. In SHRs, perinatal resveratrol supplementation mitigated the development of hypertension accompanying the improvement of NO bioavailability [155].

Several rat models of oxidative stress programming, such as maternal ADMA administration [88], high-fructose diet [96], adenine-induced CKD [99], or combined dexamethasone and TCDD exposure [105], have been used to assess the reprogramming effects of resveratrol on offspring hypertension. In a maternal CKD model, perinatal resveratrol therapy benefits on offspring’s hypertension coincided with a reduction in renal 8-OHdG expression and an increase in NO bioavailability [99]. Likewise, perinatal resveratrol therapy was found to protect combined TCDD and dexamethasone-exposure-induced hypertension in adult rat offspring and was associated with reduced renal 8-OHdG expression, decreased ADMA levels, and enhanced NO bioavailability [105]. Of note is that the protective effects of resveratrol are also associated with its prebiotic actions to alter gut microbiota [96,99]. These findings support the links between gut microbiota dysbiosis and oxidative stress that underpin the hypertension of developmental origins. 

Although resveratrol has shown beneficial effects in programmed hypertension models related to renal programming [88,96,99,105], little is known regarding its reprogramming effects in the brain, heart, and vessels. Surprisingly, the reprogramming effects of other polyphenols have not yet been explored in the hypertension models of developmental origins.

### 4.4. Melatonin

As a naturally occurring antioxidant, melatonin and its metabolites can scavenge ROS/RNS, enhance expression of antioxidant enzymes, reduce ADMA, and increase NO bioavailability [156,157]. Early-life melatonin treatment has been considered as a reprogramming strategy for many DOHaD-related diseases, including the hypertension of developmental origins [157].

Melatonin treatment in pregnancy and lactation has shown benefits for hypertension in several models of oxidative stress programming, such as maternal l-NAME exposure [46], maternal caloric restriction [84], a maternal methyl-donor-rich diet [97], and a high-fructose diet [130]. The perinatal use of melatonin has been shown to have beneficial effects via the restoration of the NO–ROS balance in a maternal caloric restriction model [84] and a high-fructose model [130]. Additionally, the protective effect of melatonin therapy accompanies diminished lipid peroxidation [46], decreased ADMA concentrations [84], reduced 8-OHdG expression [84,97], and increased NO bioavailability [84,130]. In addition to its antioxidant properties, melatonin has pleiotropically biological functions. Accordingly, the extent that the antioxidant effects of melatonin contribute to its reprogramming benefits against the hypertension of developmental origins is still ambiguous.

### 4.5. Synthetic Antioxidants

Apart from natural antioxidants, certain synthetic antioxidants have been implemented in animal models of oxidative stress programming to study the hypertension of developmental origins. *N*-acetylcysteine (NAC), a well-known thiol-containing antioxidant, has been applied in treating disorders associated with oxidative stress [158]; it works not only as a glutathione precursor but also a stable l-cysteine analog for H_2_S synthesis [158]. A significant decrease in high BP was achieved with perinatal NAC therapy in several models of oxidative stress programming, such as maternal l-NAME administration [46], maternal suramin administration [92], and dexamethasone combined with a postnatal high-fat diet [103]. The beneficial effects of NAC against offspring hypertension are related to reducing lipid peroxidation [46], increasing the glutathione level [103], decreasing renal 8-OHdG expression [103], and enhancing NO production [92].

One study reported that maternal lazaroid therapy, an inhibitor of lipid peroxidation [159], prevented hypertension in adult rat offspring born to dams receiving a protein restriction diet [87]. Another synthetic antioxidant is nuclear factor erythroid-derived 2-related factor 2 (Nrf2) activator dimethyl fumarate (DMF). Considering Nrf2 is a redox-sensing transcription factor that controls numerous genes that are involved in the management of oxidative stress [160], DMF has been used to prevent the hypertension of developmental origins in a combined dexamethasone and high-fat exposure model [104]. Notably, certain synthetic antioxidants (e.g., tempol) have been studied extensively in animal models of oxidative stress [17,161]. Nevertheless, no study has scientifically examined their ability of protecting adult progeny against hypertension programmed by oxidative stress.

As reviewed elsewhere [22,23,123], a number of currently used drugs have been reported to restore the NO–ROS balance via lowering ADMA levels. Telmisartan, rosuvastatin, glucagon-like peptide-1 receptor agonist, and epigallocatechin-3-gallate are able to reduce ADMA levels via decreased expression of the ADMA-generating enzyme. On the other hand, telmisartan, metformin, salvianolic acid A, oxymatrine, atorvastatin, and rosuvastatin can enhance the activity and/or expression of ADMA-metabolizing enzymes and thereby reduce ADMA levels [22,23]. Among them, only metformin has been tested and shown benefits against hypertension, coinciding with its ADMA-lowering action in a maternal high-fructose and post-weaning high-fat diet model [41]. 

## 5. Concluding Remarks and Perspectives

There is considerable evidence supporting the idea that oxidative stress is involved in the hypertension of developmental origins and that antioxidant therapy is a potential preventive strategy. However, there are still some unsolved aspects toward clinical translation. Considering promising data from animal studies, it seems logical to think that antioxidant therapy is a potential reprogramming strategy for the oxidative-stress-induced hypertension of developmental origins. Nevertheless, results in humans are still not conclusive, as antioxidant therapy cannot control the global rise of hypertension [12]. 

Another important aspect is that excessive antioxidant supplementation can turn oxidative stress into a reverse state, namely antioxidant stress [162]. As oxidative stress is not routinely detectable in clinical practice, oxidative stress should be monitored, and perinatal antioxidant therapy should only be used for indicated cases.

Aside from oxidative stress, the hypertension of developmental origins is associated with other core mechanisms [4,5,10,11,59,81]. The protective effects of perinatal antioxidant therapy might also be attributed to the activation of nutrient-sensing signals, rebalancing of the RAS, and altering gut microbiota. Indeed, early interventions targeting other mechanisms (e.g., AMPK activator or RAS blocker) in pregnancy have been shown to prevent progeny against the hypertension of developmental origins [59,163]. Therefore, major advances are needed in our mechanistic understanding of how oxidative stress communicates with other core mechanisms in various organs during different stages of development. 

In conclusion, oxidative stress contributes significantly to the hypertension of developmental origins. Antioxidant therapy can serve as a reprogramming strategy to prevent hypertension while awaiting clinical translation. Since the hypertension of developmental origins can be reversible, the knowledge of oxidative stress programming could aid in developing ideal antioxidants as reprogramming therapies toward reducing the global burden of hypertension.

## Figures and Tables

**Table 1:** Summary of the oxidative-stress-related hypertension of developmental origins in animal models.

Animal Models	Species/ Gender	Age at Evaluation	Mechanisms of Oxidative Stress	Programmed Organ System	Ref.
Maternal caloric restriction diet	SD rat/M	12 weeks	↑ Renal 8-OHdG expression, ↑ ADMA, ↓ NO	Kidneys	[83,84]
Maternal caloric restriction diet	Wistar rat/M	16 weeks	↑ 3-NT, ↓ NO	Vessels	[85]
Maternal caloric restriction diet	SD rat/M	6 months	↑ Xanthine-oxidase expression	Heart	[86]
Maternal protein restriction diet	Wistar rat/M	12 weeks	↑ F_2_-isoprostane, ↓ glutathione	Kidneys	[87]
Maternal L-NAME administration	SD rat/M	12 weeks	↑ Renal F_2_-isoprostane	Kidneys	[46]
Maternal ADMA administration	SD rat/M	12 weeks	↓ NO	Kidneys	[88]
Streptozotocin-induced diabetes	SD rat/M	12 weeks	↑ ADMA, ↓ NO	Kidneys	[89]
Streptozotocin-induced diabetes	SD rat/M	12 weeks	↑ Renal TBARS and 3-NT	Kidneys, vessels	[90]
Streptozotocin-induced diabetes	SD rat/M	24 weeks	↑ ROS, ↓ NO, ↓ SOD activity	Vessels	[91]
Maternal suramin administration	SD rat/M	12 weeks	↑ ADMA, ↓ NO	Kidneys	[92]
Maternal high-fructose diet	SD rat/M	12 weeks	↑ Renal 8-OHdG expression, ↓ NO	Kidneys	[93]
Maternal high-fructose diet	SD rat/M	12 weeks	↑ NADPH-oxidase expression and MDA	Brain	[94]
Maternal high-fructose diet	SD rat/M	24 weeks	↑ ROS	Spleen	[95]
Maternal plus post-weaning high-fructose diet	SD rat/M	12 weeks	↑ Renal 8-OHdG expression	Kidneys	[96]
Maternal methyl-deficient diet	SD rat/M	12 weeks	↑ Renal 8-OHdG expression	Kidneys	[97]
Maternal high methyl-donor diet	SD rat/M	12 weeks	↑ Renal 8-OHdG expression	Kidneys	[97]
Maternal adenine-induced CKD	SD rat/M	12 weeks	↑ Renal 8-OHdG expression,↑ ADMA, ↓ NO	Kidneys	[98,99]
Maternal high-fat and high-cholesterol diet	SD rat/M & F	90 days	↓ SOD activity in M; ↑ Renal MDA level in F	Kidneys	[100]
Prenatal dexamethasone exposure	Wistar rat/M & F	14 weeks	↑ NADPH-oxidase, ↓ Gpx1 expression	Adrenal glands	[101]
Prenatal dexamethasone exposure	SD rat/M	16 weeks	↓ Renal NO	Kidneys	[102]
Prenatal dexamethasone exposure plus postnatal high-fat intake	SD rat/M	16 weeks	↑ Renal 8-OHdG expression, ↓ NO	Kidneys	[103,104]
Prenatal dexamethasone plus TCDD exposure	SD rat/M	16 weeks	↑ Renal 8-OHdG expression, ↑ ADMA	Kidneys	[105]
Prenatal bisphenol A exposure plus high-fat diet	SD rat/M	16 weeks	↑ Renal 8-OHdG expression, ↑ ADMA, ↓ NO	Kidneys	[106]
Reduced uterine perfusion	SD rat/M	16 weeks	↑ Urinary F_2_-isoprostane level & renal NADPH-oxidase-dependent superoxide	Kidneys	[107]
Maternal plus post-weaning high-fat diet	SD rat/M	16 weeks	↑ Renal 8-OHdG expression	Kidneys	[108]
Maternal 1K1C model	SD rat/M	16 weeks	↑ NADPH-oxidase expression, ↑ 3-NT	Brain	[109]
Maternal angiotensin II administration	Wistar rat/M	18 weeks	↑ Renal ROS	Kidneys	[110]
Maternal high-salt diet	SD rat/M	12 weeks	↑ 3-NT, ↑ ADMA	Vessels	[111]
Maternal high-salt diet	Wistar rat/M	5 months	↑ NADPH-oxidase expression, ↑ MDA level, ↓ Antioxidant activity	Vessels	[112]
Prenatal LPSExposure	Wistar rat/M	28 weeks	↑ Renal MDA	Kidneys	[113]
Maternal di-n-butyl phthalate exposure	SD rat/M & F	18 months	↑ Renal ROS	Kidneys	[114]
Prenatal betamethasone exposure	Sheep/M	6 months	↑ 4-HNE	Brain	[115]
Prenatal betamethasone exposure	Sheep/M & F	18 months	↑ ROS, ↓ NO	Kidneys	[116]
Prenatal hypoxia exposure	SD rat/M & F	8 weeks	↑ Lipid peroxidation	Heart	[117]
Prenatal hypoxia exposure	Chicken/M & F	6 months	↓ NO	Heart, vessels	[118]
Prenatal hypoxia exposure	Sheep/M & F	9 months	↓ NO	Vessels	[119]

Studies tabulated according to animal models, species, and age at evaluation. ADMA—asymmetric dimethylarginine; 8-OHdG—8-hydroxy-2’-deoxyguanosine; TBARS—thiobarbituric acid reactive substances; 3-NT—3-nitrotyrosine; 4-NHE—4-hydroxynonenal; Gpx1—glutathione peroxidase 1; CKD—chronic kidney disease; LPS—lipopolysaccharide; SD—Sprague Dawley; M—male; F—female; L-NAME—N^G^-nitro-l-arginine methyl ester; MDA—malondialdehyde; TCDD—2,3,7,8-tetrachlorodibenzo-p-dioxin; 1K1C model—one kidney is removed and the other undergoes artery constriction.
